# Meckel's Diverticulum: Factors Associated with Clinical Manifestations

**DOI:** 10.1155/2014/390869

**Published:** 2014-04-01

**Authors:** Jeng-Jung Chen, Hung-Chang Lee, Chun-Yan Yeung, Wai-Tao Chan, Chuen-Bin Jiang, Jin-Cherng Sheu, Nein-Lu Wang

**Affiliations:** ^1^Department of Pediatrics, Mackay Memorial hospital, Hsinchu, Taiwan; ^2^Department of Pediatrics, Taipei Medical University, Taipei, Taiwan; ^3^Department of Pediatrics, Mackay Memorial Hospital, Taipei, Taiwan; ^4^Mackay Medicine, Nursing and Management College, Taipei, Taiwan; ^5^Department of Pediatric Surgery, Mackay Memorial Hospital, Taipei, Taiwan

## Abstract

*Objectives*. The purpose of this study was to investigate the clinical features of Meckel's diverticula at different ages, genders, and pathology in order to serve as a reminder to clinicians when evaluating potential cases and to help obtain an early diagnosis. *Methods*. We collected information of patients with Meckel's diverticulum diagnosed at Mackay Memorial Hospital in Taiwan from 1984 to 2009. After performing a thorough review of their charts, the clinical features of the Meckel's diverticula were analyzed according to age groups, gender, and pathology. *Result*. A total of 126 patients, with 90 males and 36 females, were enrolled in this study. Seventy-five patients were symptomatic and 51 Meckel's diverticula were found incidentally during surgery for other diseases. Among symptomatic patients, 39% of pediatric patients and 5% of adult patients had intestinal hemorrhage. Twenty-eight percent of pediatric patients and 67% of adult patients had inflammation of Meckel's diverticulum. Forty-six percent of males and 16% of females had inflammation. Conversely, 27% of males and 58% percent of females had intestinal obstruction. When Meckel's diverticulum had ectopic gastric mucosa, it tended to cause intestinal hemorrhage when the patient is young. *Conclusions*. Age, gender, and pathology affect the clinical presentations of Meckel's diverticula.

## 1. Introduction

Meckel's diverticulum (MD) is the most common congenital anomaly of the gastrointestinal tract. It was first described in 1809 [1]. MD is a remnant of the omphalomesenteric or vitelline duct [2, 3]. The prevalence ranges from 2 to 4%. Anatomically, MD is a true diverticulum containing all layers of the small intestine, arising from the antimesenteric border of the ileum and receiving its blood supply from a remnant of the vitelline artery [4]. MD involves a variety of complications, including intestinal obstruction, intussusceptions, ulceration, hemorrhage, vesico-diverticular fistulae, and tumors [4–8]. The incidence of complications differed between different articles, which analyzed only adults or pediatric populations [2, 8–11]. The purpose of this study was to investigate the clinical features of MD at different ages and genders in order to serve as a reminder to clinicians when evaluating potential cases and to help obtain an early diagnosis.

## 2. Methods 

### 2.1. Patients and Medical Records

We retrospectively reviewed the charts of all patients with MD who were managed at Mackay Memorial Hospital in Taiwan from 1984 to 2009. The diagnosis of MD was made according to surgical and pathologic findings. Data extracted from the charts included gender, the age at presentation, clinical manifestations, and the surgical and pathologic findings.

### 2.2. Definition of the Age Groups and Clinical Presentations

According to the age at presentation, these enrolled patients were divided into two groups: pediatric group (<18 yr) and adult group (>18 yr). The clinical manifestations were categorized into intestinal hemorrhage, inflammation, and intestinal obstruction. Intestinal hemorrhage was diagnosed when patients had gross or microscopic rectal bleeding. The diagnosis of inflammation was made by clinical manifestations with local abdominal pain or signs of peritonitis and surgical findings, including gross erythematous change, pus or gangrene formation, or perforation, and pathologic findings. Intestinal obstruction was defined when MD was presented with abdominal pain with bilious vomiting, and intussusception, adhesion ileus, hernia, torsion, or other mechanical intestinal obstructions were found during surgery.

### 2.3. Statistical Analysis

The clinical features and pathologic findings of MD were analyzed according to the different age groups and genders by software PASW Statistics 18. The logistic regression and adjusted odds ratios were used for statistical analysis. For logistic regression models, all adjusted odds ratios (OR) are significant if 95% confidence intervals (CI) do not include 1.00.

## 3. Results

### 3.1. Demographic Data

A total of 126 patients, with 90 males and 36 females, were enrolled in this study, including 82 pediatric patients and 44 adult patients. Seventy-five patients were symptomatic (56 males and 19 females). Fifty-one MD were found incidentally during surgery for other diseases, including appendicitis, hernia, meconium peritonitis, intestinal perforation, malrotation with midgut volvulus, trauma, congenital gastrointestinal tract anomalies, and cancer. Among the asymptomatic patients, 34 were males and 17 were females.

### 3.2. Clinical Manifestations of Symptomatic Patients

Fifty-four pediatric patients and 21 adult patients became symptomatic because of complication of MD. The complications of symptomatic MD (*n* = 75) included intestinal hemorrhage (*n* = 22), inflammation (*n* = 29), and intestinal obstruction (*n* = 26). The causes of intestinal obstruction were adhesion ileus (*n* = 8), bowel torsion (*n* = 6), and intussusceptions (*n* = 12). Among the patients with intestinal hemorrhage, two males did not receive a surgical intervention initially. One patient had rectal bleeding when he was 11 years old and received surgery because of an intussusception 3 years later. The other one presented with an intestinal hemorrhage when the patient was 3 years old and then had diverticulitis two years later. One inflamed MD contained a fish bone. The youngest symptomatic patient was diagnosed because of an intestinal hemorrhage when he was 17 days old, and the oldest patient had diverticulitis when he was 76 years old.

### 3.3. Clinical Manifestations by Age, among Symptomatic Patients with Meckel's Diverticulum

Among 75 symptomatic MD, the patient numbers of different clinical manifestations were recorded and compared according to different age groups (Table 1). In the pediatric group, intestinal hemorrhage (39%) and intestinal obstruction (37%) are more common than inflammation (28%). But in the adult group, two-thirds of MD presented as inflammation. Besides, intestinal hemorrhage is rare in the adult group and only one MD was found due to rectal bleeding. When comparing between different age groups, intestinal hemorrhage occurred more common in the pediatric group than in the adult group. Conversely, the adult patients had higher chance to have inflammation of MD than the pediatric patients did. The prevalence of intestinal obstruction was not statistically significant between the adult group and pediatric group.

### 3.4. Clinical Manifestations by Gender, among Symptomatic Patients with Meckel's Diverticulum

Among 75 symptomatic MD, the patient numbers of different clinical manifestations were also recorded and compared according to gender (Table 2). Of male patients, the most common complication was inflammation of MD (46%), which was followed by intestinal hemorrhage (30%) and intestinal obstruction (27%). Of female patients, intestinal obstruction (58%) was the main clinical manifestation, and 26% had intestinal hemorrhage and 16% had inflammation. When comparing between different genders, inflammation of MD occurred more common in males than in females, and females were at higher risk to have intestinal obstruction than males were. But gender did not affect the prevalence of intestinal hemorrhage.

### 3.5. Pathologic Findings

Pathologically, among total 126 MD, 35 contained ectopic tissues, including gastric mucosa (*n* = 24), ectopic pancreas (*n* = 5), or both (*n* = 6). One patient with a gastric mucosa-containing MD initially presented with an intestinal hemorrhage and subsequently had complication with diverticulitis later. One young male had rectal bleeding when he was 11 years old and received surgery because of an intussusception 3 years later, but no ectopic gastric mucosa was found pathologically in his MD.

Among 30 gastric mucosa-containing MD, 25 (83%) were found in the pediatric group and 5 (17%) were found in the adult group (*P* = 0.017). The prevalence of gastric mucosa-containing MD is similar between males and females (30% and 33% resp.). Besides, 26 of 30 (87%) gastric mucosa-containing MD were symptomatic, and 59 of 96 (61%) MD that did not contain gastric mucosa were found when becoming symptomatic. Gastric mucosa-containing MD had higher chance to become symptomatic than those without gastric mucosa, and this was statistically significant (OR 6.24; 95% CI 2.02–19.23; *P* = 0.001). Among 4 asymptomatic gastric mucosa-containing MD, only one was not found until the patient was an adult when surgery was performed for other diseases.

Among 75 symptomatic MD, clinical manifestations were summarized according to pathology (Table 3). Gastric mucosa-containing MD had higher chance to bleed than those without gastric mucosa, and it was statistically significant. Conversely, MD that did not contain gastric mucosa tended to be complicated with inflammation and intestinal obstruction.

Of 5 MD that contained an ectopic pancreas without gastric mucosa, 2 had intestinal hemorrhage, one was complicated with intestinal obstruction, and 2 were asymptomatic.

## 4. Discussion

Meckel's diverticulum is the most common anatomic variant of the alimentary tract. Most cases of MD are connected to the ileum within 100 cm of the ileocecal valve [4, 12]. The diverticulum is on the antimesenteric side of the ileum, and the arterial blood supply and venous drainage are through remnants of the embryologic omphalomesenteric vessels. Estimated prevalence is 2~4% of the population [2, 13–15]. Even no difference in the prevalence of MD between males and females, symptomatic MD has a male predominance with a male-to-female ratio ranging from 2 : 1 to 5 : 1 [2, 13, 16, 17]. The total lifetime rate of complications is estimated to be around 4% [7, 18]. The incidence of symptomatic MD is more common in children than adults [13, 17]. And the risk of complications decreases to zero in old age [7]. In this study, the male-to-female ratio was 2.5 : 1. The youngest symptomatic patient was diagnosed because of an intestinal hemorrhage when the patient was 17 days old and the oldest patient had diverticulitis when the patient was 76 years old. Among symptomatic MD, 72% of patients were younger than 18 years old and only 28% of patients are adults. MD tended to become symptomatic before the patients grew up.

MD presents with varied clinical manifestations. Complications of MD often result from ectopic tissues or bands and cause intestinal hemorrhage, diverticulitis with or without peritonitis, and intestinal obstruction. According to previous articles, the clinical presentations may differ between pediatric population and adults. In the pediatric population, intestinal hemorrhage and obstruction were more common than diverticulitis [13, 19]. In adults, intestinal obstruction was the most common complication [7, 8]. In this study, intestinal hemorrhage and obstruction were the most common clinical presentation in the pediatric population, while inflammation was the most common clinical presentation in adults. It was also noted that intestinal hemorrhage is uncommon in adults.

In this study, the clinical presentations were analyzed according to different age groups and genders.

### 4.1. Intestinal Hemorrhage

Pathologically, MD is lined mainly by the ileal mucosa. However, MD may contain ectopic tissues, including ectopic gastric, duodenal, colonic, pancreatic or endometrial mucosa, Brunner's glands, and hepatobiliary tissue [5]. The incidence of ectopic gastric tissue in MD in symptomatic patients was previously reported to be 20–80% [19–21]. Intestinal hemorrhage resulted from ileal mucosal ulceration adjacent to acid-producing ectopic gastric mucosa [2]. In children, 27%~56/% of MD presented with intestine hemorrhage [13, 19, 22]. For adults, the incidence of intestinal hemorrhage was 8%~38% [9, 10].

In this study, among total 126 patients, gastric mucosa-containing MD had higher chance to become symptomatic than those without gastric mucosa. When gastric mucosa-containing MD became symptomatic, it tended to present as intestinal hemorrhage rather than inflammation or intestinal obstruction. Conversely, when MD did not contain gastric mucosa, it tended to be complicated with inflammation and intestinal obstruction but not intestinal hemorrhage. Besides, gastric mucosa-containing MD were found more frequently in the pediatric group than in the adult group. It seems that gastric mucosa-containing MD tended to bleed easily. Therefore, it became symptomatic and complicated with intestinal hemorrhage when the patient is young. In pediatric population, lower gastrointestinal hemorrhage can be caused by numerous diseases such as necrotizing enterocolitis, malrotation with volvulus, anal fissure, intussusceptions, infectious colitis, and Meckel's diverticulum. Proper history taking and physical examination help differentiate the possible diagnoses. When infants or children had rectal bleeding without other symptoms such as fever, diarrhea, or abdominal pain, MD should be considered and further studies that detect ectopic gastric mucosa are indicated.

### 4.2. Inflammation

MD can become symptomatic when inflamed, which results in diverticulitis, perforation, and even peritonitis. Inflamed MD and appendicitis share similar clinical symptoms and signs, including fever, nausea, vomiting, tenderness to palpation, and rebound pain. The mechanism of inflammation of MD was also thought to be similar to appendicitis, which is caused by obstruction of the lumen leading to inflammation and even perforation and peritonitis [23].

In this study, the odds of inflammation of MD in males were 4.6 times than in females. Similar to appendicitis, inflammation of MD has a male predominance. Inflammation of MD also occurred more frequently in adults than in children. The odds of inflammation of MD in adult were 5.2 times more than the pediatric population. Inflammation of MD was the main clinical presentation in adults. Because of the similar location within the abdomen, clinical manifestation, the male tendency, and age of onset (mainly in the teenagers and adults), inflammation of MD was undistinguishable from acute appendicitis clinically [2]. Preoperation diagnoses of 16 patients in this study were appendicitis. If the preoperation diagnosis is appendicitis, but the appendix was found to be normal when surgery took place, exploration for MD should be performed [23].

### 4.3. Intestinal Obstruction

MD can cause intestinal obstruction, which presents with abdominal pain, nausea, vomiting, and distension. Intestinal obstruction can be secondary to (1) volvulus around the vitelloumblical cord, (2) intussusception, (3) inflammation with adhesion, (4) band between MD and mesenterium, and (5) internal hernia or Littre hernia [23–28].

The incidence differs according to different studies and articles. According to Yahchouchy, obstruction is the most common presenting symptom in the adult population, occurring in almost 40% of patients [2]. But Bemelman et al. thought that intestinal obstruction occurred mainly in patients under the age of 10 [11]. In this study, among symptomatic patients, 37% of children and 28% of adults presented with intestinal obstruction. The difference with regard to incidence was not statistically significant.

In this study, females had a higher incidence of intestinal obstruction than males (58% versus 27%; *P* = 0.017). Even though the reason is unclear, the gender trend may be important to female patients. Unlike males, females can be pregnant. And intestinal obstruction is a rare complication of pregnancy with significant maternal and fetal mortality [29]. The presence of MD as the cause of obstruction during pregnancy is extremely rare. It requires the extirpation of the diverticulum and bowel resection in 23% of cases and fetal mortality rate may be around 20% [30]. A high index of suspicion is required so that timely surgical intervention can be performed to minimize the risks to the mother and fetus [31].

## 5. Conclusion

The clinical presentations of MD differed by age and gender. Intestinal hemorrhage occurred more frequently in the pediatric population and in cases of MD that contained ectopic gastric mucosa. When a pediatric patient presents rectal bleeding without other symptoms or signs of infection or bowel obstruction, further studies that detect ectopic gastric mucosa of MD should be considered. Conversely, inflammation of MD was found more often in males and adults. Inflammation of MD and acute appendicitis had similar clinical presentations, and they were undistinguishable clinically. Intestinal obstructions occurred more frequently in females than in males. Early diagnosis of complicated MD in pregnant women is very important because it can cause maternal and fetal mortality.

## Figures and Tables

**Table 1:** Clinical manifestations by age, among symptomatic patients with Meckel's diverticulum.

	Pediatric group *n* = 54 (%)	Adult group *n* = 21 (%)	Odds ratio (95% CI)	*P* value
Intestinal hemorrhage	21 (39%)^†‡^	1 (5%)	12.72 (1.59–102.3)	0.017
Inflammation	15 (28%)^†^	14 (67%)	0.19 (0.07–0.57)	0.003
Intestinal obstruction	20 (37%)^‡^	6 (28%)	1.47 (0.49–4.4)	0.49

^†^One patient had intestinal hemorrhage initially and diverticulitis later.

^‡^One patient had intestinal hemorrhage initially and intussusception later.

**Table 2:** Clinical manifestations by gender, among symptomatic patients with Meckel's diverticulum.

	Male *n* = 56 (%)	Female *n* = 19 (%)	Odds ratio (95% CI)	*P* value
Intestinal hemorrhage	17 (30%)^†‡^	5 (26%)	1.22 (0.38–3.93)	0.738
Inflammation	26 (46%)^†^	3 (16%)	4.62 (1.21–17.66)	0.025
Intestinal obstruction	15 (27%)^‡^	11 (58%)	0.27 (0.09–0.79)	0.017

^†^One patient had intestinal hemorrhage initially and diverticulitis later.

^‡^One patient had intestinal hemorrhage initially and intussusception later.

**Table 3:** Clinical manifestations by pathology, among symptomatic patients with Meckel's diverticulum.

	Gastric mucosa(+) *n* = 26 (%)	Gastric mucosa(−) *n* = 49 (%)	Odds ratio (95% CI)	*P* value
Intestinal hemorrhage	14 (54%)^†^	8 (16%)^‡^	5.98 (2.03–17.62)	0.001
Inflammation	7 (27%)^†^	22 (45%)	0.42 (0.16–1.27)	0.132
Intestinal obstruction	6 (23%)	20 (41%)^‡^	0.44 (0.15–1.28)	0.129

^†^One gastric mucosa-containing MD complicated with intestinal hemorrhage initially and diverticulitis later.

^‡^One MD that did not contain gastric mucosa complicated with intestinal hemorrhage initially and intussusception later.
